# Yeast fermentate prebiotic improves intestinal barrier integrity during heat stress by modulation of the gut microbiota in rats

**DOI:** 10.1111/jam.14361

**Published:** 2019-07-09

**Authors:** H.A.G. Ducray, L. Globa, O. Pustovyy, E. Morrison, V. Vodyanoy, I. Sorokulova

**Affiliations:** ^1^ Department of Anatomy, Physiology and Pharmacology Auburn University Auburn AL USA

**Keywords:** gut microbiota, heat stress, rats, *Saccharomyces cerevisiae* fermentate, tight junction proteins

## Abstract

**Aims:**

To evaluate efficacy of *Saccharomyces cerevisiae* fermentate prebiotic (EH) in protection of intestinal barrier integrity in rats during heat stress, to analyze the impact of heat stress and preventive treatment with EH on the structure of the gut microbiota.

**Methods and Results:**

Two groups of rats were treated orally with EH or phosphate‐buffered saline for 14 days. On day 15, half of the rats in each group were exposed to heat stress conditions, while control animals were kept at room temperature. Histological and Western blot analyses of the intestine, culture‐based microbiological analysis and high‐throughput 16S rRNA sequencing for the gut microbiota were performed for each rat. Exposure of animals to heat stress conditions resulted in inhibition of tight junction (TJ) proteins expression, decrease of Paneth and goblet cells, decrease of beneficial and increase of pathogenic bacteria. Oral treatment of rats with EH before stress significantly prevents these adverse effects by elevation of the gut beneficial bacteria, particularly butyrate‐producing bacteria.

**Conclusions:**

Essential effect of EH in protection of intestinal barrier integrity during heat stress is connected with beneficial modulation of the gut microbiota.

**Significance and Impact of the Study:**

Our results will contribute to the development of new approaches to prevention of heat stress‐related complications.

## Introduction

Heat stress, as other types of stress, seriously impacts gastrointestinal physiology, which result in intestinal ulceration, development of irritable bowel syndrome and inflammatory bowel disease (Soderholm and Perdue, 2001; Yu *et al.,* 2010). It was shown that stress significantly affects intestinal barrier function resulted in gut permeability and systemic inflammation (Lambert, 2009). One of the mechanisms connecting stress and gastrointestinal diseases is stress‐induced effects on mucosal barrier function (Konturek *et al.,* 2011). Intestinal barrier function is the ability to control uptake across the mucosa and to protect the inner environment from potentially harmful compounds present in the intestinal lumen. This barrier is achieved by the intracellular junctional complexes: tight junctions (TJ), adherens junctions, gap junctions and desmosomes (Suzuki, 2013). The TJ are the apical‐most junctional complex, responsible for sealing the intercellular space. They act as a primary barrier to the diffusion of solutes through the intercellular space. The main types of transmembrane proteins in TJ are occludin and claudins, which link adjacent enterocytes (Ohland and MacNaughton, 2010). Zonula occludens (ZO) proteins are important intracellular TJ proteins that link the transmembrane TJ proteins: claudins, occludin and junctional adhesion molecules (JAM) to the actomyosin cytoskeleton (Grootjans *et al.,* 2010). Disruption of the intestinal TJ barrier, induces activation of the mucosal immune system and inflammation, and can act as a trigger for the development of intestinal and systemic diseases (Suzuki, 2013). Various factors may cause destabilization of TJ proteins: enteric pathogens and their toxins, anti‐inflammatory drugs, alcohol (Groschwitz and Hogan, 2009). Heat stress was shown to disrupt intestinal barrier function (Hall *et al.,* 2001) and to change the expression of TJ proteins (Xiao *et al.,* 2013). Usually, the effect of heat stress on TJ proteins is assessed *in vitro* in epithelial cell monolayers. Recently, Pearce *et al. *(2013) showed changes in the TJ proteins composition in pigs, exposed to heat stress, but authors did not propose approaches to prevent/reduce this adverse effect of heat stress. It was found that Paneth and goblet cells are critical for maintenance of intestinal barrier (Vaishnava *et al.,* 2008; Bevins and Salzman, 2011; Johansson and Hansson, 2016). Goblet cells are responsible for production of mucins, forming the basic skeleton of mucus layer, which serves as a first line of innate defence. Paneth cells produce different antimicrobial compounds essential for control intestinal barrier and limit bacterial penetration to host tissues. Keeping the integrity of the intestinal barrier is a key for intestinal homeostasis and overall for the health status of the host. It was shown that microbiota and its metabolites can regulate the gut barrier function (Kelly *et al.,* 2015; Jakobsson *et al.,* 2015). Exposure to various types of stress results in significant changes in the composition of the gut microbiota and associated complications (Bailey *et al.,* 2011). Prebiotics and probiotics have been proposed as a promising approach to normalize microbiota and, as a result to improve intestinal barrier function (Russo *et al.,* 2012; Wilms *et al.,* 2016). Our previous study showed that fermentate of *Saccharomyces cerevisiae* was very effective in prevention of heat stress‐related complications in rats (traumatic changes of the gut morphology, elevation of serum lipopolysaccharides, pathology of erythrocytes) (Ducray *et al.,* 2016). These beneficial effects of yeast fermantate are due to prebiotic activity of this product, previously confirmed *in vitro* (Possemiers *et al.,* 2013) and in clinical trials (Pinheiro *et al.,* 2017). We hypothesize that EH can protect the gut microbiota and improve intestinal barrier function during heat stress conditions, thus preventing adverse effects of heat. The main objectives of this study were to evaluate efficacy of EH in protection of intestinal barrier integrity during heat stress, to analyze the impact of heat stress and preventive treatment with EH on the structure of the gut microbiota.

## Materials and methods

### Ethics statement

All animal procedures were approved by the Auburn University Institutional Animal Care and Use Committee (protocol number 2016‐2853). The study was performed in accordance with the recommendations in the Guide for the Care and Use of Laboratory Animals of the National Institutes of Health.

### Animals

Adult male Sprague–Dawley  rats weighing 250–300 g were purchased from Harlan Laboratories (Indianapolis, IN). Animals were housed under specific pathogen free conditions with a 12‐h light/dark cycle at (20 ± 1)°C, and were provided with standard food (2018 Teklad Global 18% Protein Rodent Diet; Harlan) and water *ad libitum.*

### *Saccharomyces cerevisiae* fermantate

The powder form of *S. cerevisiae* fermentate (EH) was provided by the manufacturer (Embria Health Sciences, Ankeny, IA). EH is rich in yeast cell fragments and various metabolites, including polyphenols, polysaccharides such as beta glucan, trace minerals, amino acids and peptides (Pinheiro *et al.,* 2017). Before oral treatment of rats yeast fermentate was diluted in phosphate‐buffered saline (PBS) at the rate 7 mg kg^−1^ of animal weight in 1 ml of PBS.

### Antibodies

Primary rabbit polyclonal antibodies against zonula occludence (ZO‐1) (#40‐2200), occludin (#40‐4700), mouse anti‐claudin‐1‐monoclonal antibody (#37‐4900) and beta‐Actin Loading Control antibody (# MA5‐15739) were from ThermoFisher Scientific (Waltham, MA), rabbit polyclonal antibodies against JAM‐A (#ab125886) were from Abcam (Cambridge, MA). IRDye 800CW goat anti‐rabbit (#926‐32211) and IRDye 800CW goat anti‐mouse (#926‐32210) secondary antibody were from LiCor (Lincoln, NE).

### Experimental design

**Figure 1:**
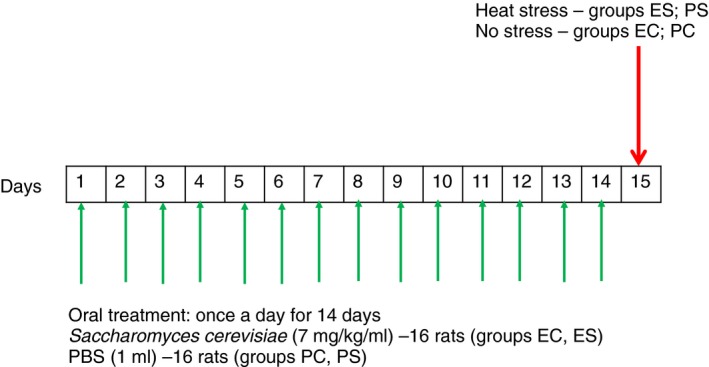
Experimental design. Two groups of rats (16 rats in each group) were treated by oral gavage with 1 ml of *Saccharomyces cerevisiae* fermentate prebiotic (EH) or with 1 ml of PBS once a day for 14 days (Fig. 1). On day 15, rats in each group were subdivided (eight rats in each group): PC—control (PBS/room temperature), EC—control prebiotic (EH/room temperature), PS—PBS + stress (PBS/45°C) and ES—prebiotic + stress (EH/45°C). Animals from group PS and ES were exposed for 25 min to heat stress conditions (45°C, relative humidity 55%) in a climatic chamber. Control animals (groups PC and PS) were kept at room temperature. [Colour figure can be viewed at http://wileyonlinelibrary.com]

Animal model of heat stress was successfully used in our previous study (Ducray *et al.,* 2016). Briefly, two groups of male Sprague–Dawley rats weighing 250–300 g (16 rats in each group) were treated by oral gavage with 1 ml of yeast fermentate prebiotic (EH group) or with 1 ml of PBS (PBS group) once a day for 14 days (Fig1). On day 15, rats in each group were subdivided (eight rats in each group): PC—control (PBS/room temperature), EC—control prebiotic (EH/room temperature), PS—PBS + stress (PBS/45°C) and ES—prebiotic + stress (EH/45°C). Animals from group PS and ES were exposed for 25 min to heat stress conditions (45°C, relative humidity 55%) in a climatic chamber (Environmental Chamber 6020‐1; Caron, Marietta, OH). Control animals (groups PC and EC) were kept at room temperature. Rectal temperature was measured in each rat before and immediately after the experiment. Animals were allowed to stand 4 h at room temperature after the experiment, because it was showed that maximal effect of stress on epithelial function was 4 h after exposure to stress conditions (Soderholm *et al.,* 2002; Zareie *et al.,* 2006). Four hours after the stress experiments, rats were anesthetized with isoflurane (2–4%) and euthanized by rapid decapitation. Samples of small intestine from each rat were taken for morphological analysis and Western blot. Faecal matter from the colon was immediately placed in anaerobic broth for culture‐based microbiological analysis. For 16S rRNA sequencing of the gut microbiota faecal samples were placed at −80°C until the experiment.

### Histological analysis

Samples of the small intestine were prepared as it was previously described (Ducray *et al.,* 2016). Briefly, samples were fixed in Bouin’s fixative (Electron Microscopy Sciences, Hatfield, PA) embedded in paraffin, sectioned at 6 μm, slide mounted, haematoxylin and eosin stained, and cover‐slipped. Haematoxylin‐eosin staining was performed according to the standard protocol (Stevens, 1990).

### Counting of goblet cells

Four sections from each rat were stained as previously described (Trevizan *et al.,* 2016). Briefly, sections were subjected to a series of deparaffinization, stained with Alcian Blue (Electron Microscopy Sciences) for 30 min, washed with tap and distilled water, treated with 0·5% periodic acid (Electron Microscopy Sciences), washed with distilled water for 2 min, stained with Schiff’s Reagent (Electron Microscopy Sciences) for 20 min, washed with tap water for 5 min, stained with haematoxylin (1 min), washed with tap water for 2 min, dehydrated, cleared in HemoDi (Fisher Scientific, Pittsburgh, PA) and mounted in Eukitt Mounting Medium (Electron Microscopy Sciences). Eight images from each section were taken with a digital camera (Pro series 3CCD camera) coupled to an optical microscope (Olympus BX50, Microscope Central, Feasterville, PA) with a 20x objective. The number of goblet cells presented in 0·96 mm^2^ in the mucosa of each animal were quantified using ImagePro Plus software (Media Cybernetics, Rockville, MD).

### Paneth cells counting

Phloxine‐tartrazine technique were used to analyze Paneth cells, as previously reported (Di Sabatino *et al.,* 2008). Briefly, sections were treated with alum haematoxylin (5 min), washed with tap water (5 min), stains in phloxine B‐ calcium carbonate (Electron Microscopy Sciences) for 20 min, rinsed in tap water, blot dried, stained saturated solution of tartrazine saturated cellosolve (Electron Microscopy Sciences) for 10 min, rinsed in 95% alcohol, dehydrated in absolute alcohol, cleared in HemoDi (Fisher Scientific) and mounted in Eukitt Mounting Medium (Electron Microscopy Sciences). The amount of Paneth cells were counted for each sample using a high resolution microscope system (Vainrub *et al.,* 2006). Four sections from each rat were analyzed.

### SDS‐PAGE and Western Blotting

Intestinal tissues were snap‐frozen in liquid nitrogen and kept at −80°C until study. Tissues were homogenized using T‐PER Reagent with Protease Inhibitor Cocktail (Thermo Scientific, Rockford, IL). Samples were centrifuged at 15 000 ***g*** for 30 min at 4°C and supernatants were collected. A protein assay (Bio‐Rad, Hercules, CA) was conducted to determine the protein concentration for each sample. An equal amount of proteins (50 µg) were separated by SDS–PAGE (10%) and transferred to nitrocellulose membranes. The membranes were blocked for 1 h in Odyssey blocking buffer (LiCor) and incubated overnight at 4°C with primary antibodies against β‐actin, claudin, occludin, ZO‐1 or JAM‐A proteins. The membranes were washed with PBS/0·1% Tween‐20 three times and incubated with goat anti‐rabbits IRDye 800CW secondary antibodies for 1 h, then washed with PBS/0·1% Tween‐20 four times. Membranes were imaged by LiCor Odyssey scanner, and blots were analyzed by Image Studio 2.0 analytical software (LiCor). The procedure was repeated at least four times for each protein. Bands were standardized to the density of actin and were represented as a ratio of each protein to actin.

### Analysis of the gut microbiota

#### Culture‐based microbiological study

Determination of the gut microbiota was performed according to methods described previously (Sudo *et al.,* 2004; Nishino *et al.,* 2013). Faecal matter was removed from the colon of each rat using sterile technique, placed in sterile preweighted tubes with anaerobic broth, weighted and vortexed until homogenous. Serial 10‐fold dilutions from 10^−1^ to 10^−7^ were prepared and from the appropriate dilution, a 0·1 ml aliquot was then spread on four plates with different media: Anaerobic Basal Agar (Alfa Aesar, Tewksbury, MA) for total anaerobic bacteria; Brain Heart Infusion Agar (Hardy Diagnostic, Santa Maria, CA) for total aerobes; Blood agar (Hardy Diagnostic) for haemolytic bacteria; Violet Red Bile Agar (Hardy Diagnostic) for *Enterobacteriaceae* *;* Bifidobacterium agar (HiMedia Laboratories, West Chester, PA) for *Bifidobacterium*; Difco Lactobacilli MRS agar (Becton Dickinson, Sparks, MD) for *Lactobacillus;* BBL Mannitol Salt agar (Becton Dickinson) for *Staphylococcus;* Brucella agar with hemin and vitamin K1 (HiMedia Laboratories) for *Bacteroides*; Reinforced Clostridial Medium (Hardy Diagnostic, Santa Maria, CA) for *Clostridium*; Sabouraud agar (HiMedia Laboratories) for yeasts. For isolation of anaerobic bacteria plates were placed in an anaerobic chamber in a microaerophilic environment generated by a GasPak EZ Anaerobe Container System (Becton Dickinson and Co). All plates were incubated at 37°C and colonies were counted after incubation for 24 h for aerobes and 48 h for anaerobes. The number of colony‐forming units per gram of faecal matter was calculated. Bacterial cultures and yeasts were identified by morphology of colonies, microscopical analysis of cells’ morphology, Gram staining, formation of spores, aerobic and anaerobic growth, as it was recommended elsewhere (Benno and Mitsuoka, 1992; Sudo *et al.,* 2004).

#### High‐throughput 16S rRNA sequencing for the gut microbiota

Faecal samples were submitted to MR DNA (Shallowater, TX) for DNA isolation and sequencing. Genomic DNA was isolated from samples using a QIAamp DNA stool mini kit (Qiagen, Germantown, MD) following the manufacturer's instructions. The purified DNA was eluted from the spin filter using 50 μl of solution C6 and stored at −20°C until PCR amplification.

Amplicon sequencing using next generation technology (bTEFAP) was originally described by Dowd *et al. *(2008). The 16s rRNA V1‐V3 primers, 27F AGRGTTTGATCMTGGCTCAG and 519R GTNTTACNGCGGCKGCTG, were utilized to evaluate the microbial ecology of each sample on the MiSeq with methods via the bTEFAP DNA analysis service. Each sample underwent a single‐step 30 cycle PCR using HotStarTaq Plus Master Mix Kit (Qiagen, Valencia, CA) were used under the following conditions: 94°C for 3 min, followed by 28 cycles of 94°C for 30 s; 53°C for 40 s and 72°C for 1 min; after which a final elongation step at 72°C for 5 min was performed. Following PCR, all amplicon products from different samples were mixed in equal concentrations and purified using Agencourt Ampure beads (Agencourt Bioscience Corporation, MA). Samples were sequenced utilizing the Illumina MiSeq chemistry following manufacturer’s protocols. The Q25 sequence data derived from the sequencing process was processed using a proprietary analysis pipeline (MR DNA, Shallowater, TX). Sequences were depleted of barcodes and primers then short sequences <200 bp were removed, sequences with ambiguous base calls removed, and sequences with homopolymer runs exceeding 6bp removed. Sequences were then denoised and chimeras removed. Operational taxonomic units (OTUs) were defined after removal of singleton sequences, clustering at 3% divergence (97% similarity). OTUs were then taxonomically classified using blastn against a curated NCBI database.

### Bioinformatics analysis

Statistical analysis of sequence results was performed using a variety of computer packages including XLstat, NCSS 2007, ‘R’ and NCSS 2010. Significance reported for any analysis is defined as *P* < 0·05.

### Statistical analysis

All results were presented as a mean and standard deviation. The difference between groups was analyzed by the one‐way anova, followed by the Bonferroni test (Baurhoo *et al.,* 2007; Possemiers *et al.,* 2013). The significance level was set at 0·05 to define statistical significance. Statistical calculations and graph plotting were carried out using Microcal™ Origin ver. 9.0 (Northhampton, MA) and 2010 Microsoft Excel.

## Results

### Body temperature

Body temperature of rats, exposed to heat stress conditions (PS and ES groups) significantly increased. The mean body temperature was 37·55 ± 0·16°C before and 40·98 ± 0·43°C immediately after stress (*P* < 0·05) in PS group and 37·66 ± 0·73°C before and 40·50 ± 0·60°C after (*P* < 0·05) in ES group. No change in body temperature of control rats, not exposed to stress (PC and EC groups) was found.

### Tight junction proteins expression

**Figure 2:**
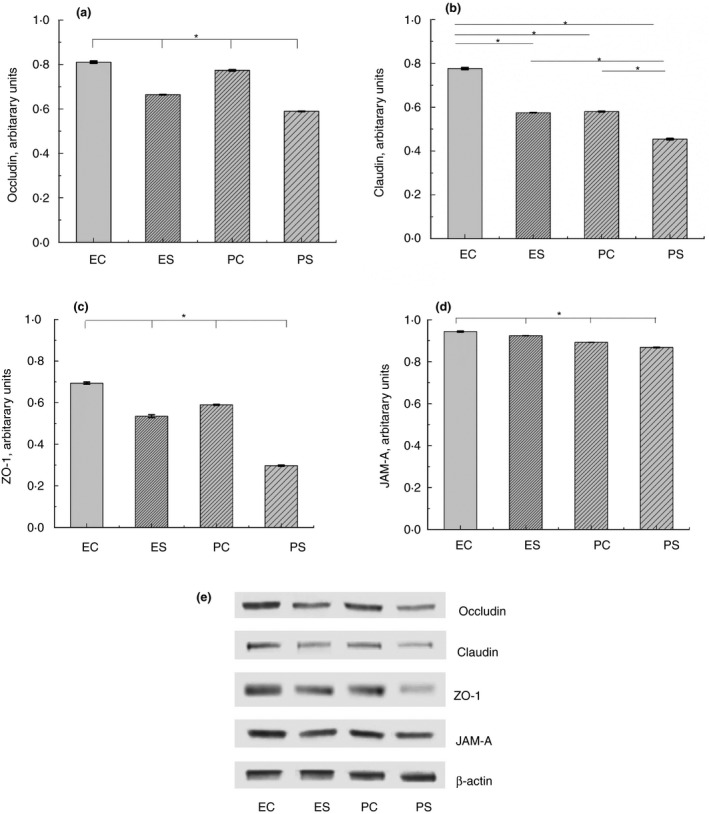
Expression of TJ proteins in the intestine of rats from different experimental groups: ES—rats were orally gavaged with *Saccharomyces cerevisiae* fermentate and exposed to heat stress; EC—rats were orally gavaged with *Saccharomyces cerevisiae* fermentate and kept at room temperature; PS—rats were orally gavaged with PBS and exposed to heat stress; PC—rats were orally gavaged with PBS and kept at room temperature; *P < 0·05.

Expression of TJ proteins (occludin, claudin, ZO‐1 and JAM‐A) in the intestine of all rats was analyzed by Western blot. Expression of all tested proteins was significantly depressed in animals from PS group in comparison with other groups (*P* < 0·05) (Fig2). Pretreatment with EH before exposure to heat stress (group ES) resulted in significantly increased level of all proteins in comparison with PS group (*P* < 0·05), though lower in comparison with EC group.

### Paneth cells number

**Figure 3:**
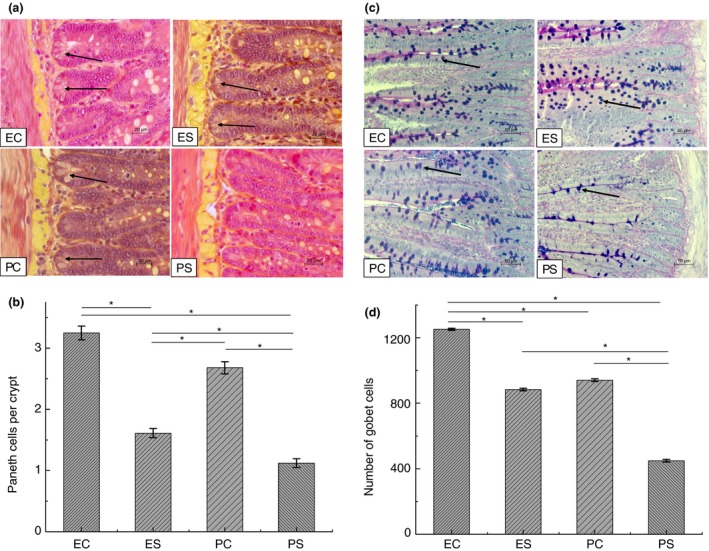
Paneth and goblet cells in the intestine of rats from different experimental groups. (a) Histological samples of the small intestine were stained with phloxine‐tartrazine to analyze Paneth cells, scale bar = 20 µm; arrows show Paneth cells; (b) number of Paneth cells, *P < 0·05; (c) Alcian blue staining of goblet cells in histological samples of the small intestine, scale bar = 50 µm; Arrows show goblet cells; (d) Goblet cells number, *P < 0·05. ES—rats were orally gavaged with *Saccharomyces cerevisiae* fermentate and exposed to heat stress; EC—rats were orally gavaged with *Saccharomyces cerevisiae* fermentate and kept at room temperature; PS—rats were orally gavaged with PBS and exposed to heat stress; PC—rats were orally gavaged with PBS and kept at room temperature. [Colour figure can be viewed at http://wileyonlinelibrary.com]

The number of Paneth cells in rats, exposed to heat stress (groups PS and ES), was significantly lower in comparison with control groups (PC and EC). Supplementation of rats with EH before heat stress (ES group) prevented the loss of Paneth cells in comparison with rats, pretreated with PBS (PS group) (1·61 ± 0·07 and 1·12 ± 0·07 accordingly, *P* < 0·05) (Fig. 3a,b).

### Number of goblet cells

The number of goblet cells was significantly decreased in rats from PS group in comparison with control rats (group PC) (448·8 ± 8·4 and 940·8 ± 8·4 accordingly, *P* < 0·05). Goblet cell count in intestine of heat stressed rats pretreated with EH (group ES) was lower than in nonstressed rats from EC group (883·8 ± 7·8 and 1251 ± 6·6 accordingly, *P* < 0·05), but significantly higher than in animals pretreated with PBS before exposure to heat stress (PS group) (Fig. 3c,d). Treatment of control rats with EH (EC group) resulted in significant elevation of goblet cells in comparison with control PC group.

### Culture‐based analysis of the gut microbiota

**Figure 4:**
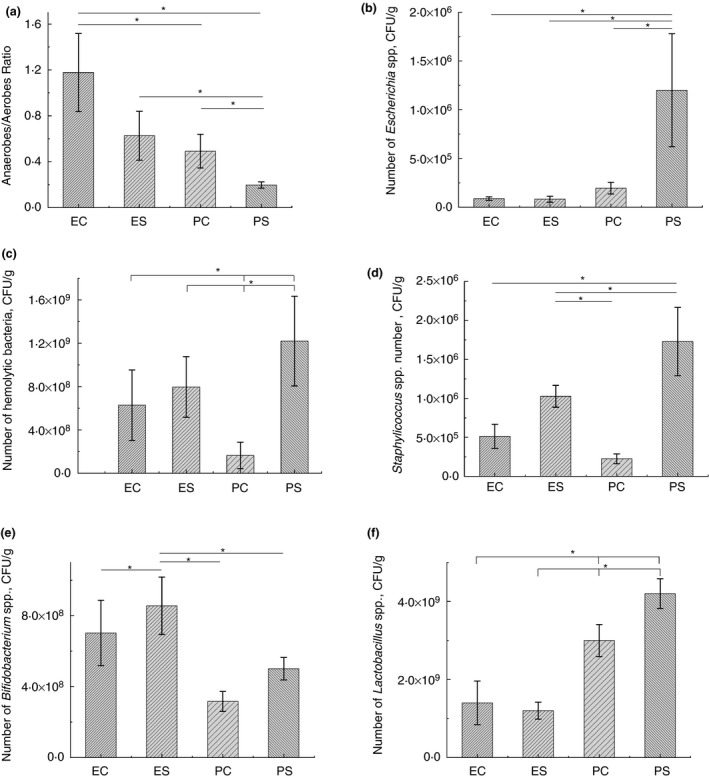
Analysis of the gut microbiota of rats by a culture‐based method. ES—rats were orally gavaged with *Saccharomyces cerevisiae* fermentate and exposed to heat stress; EC—rats were orally gavaged with *Saccharomyces cerevisiae* fermentate and kept at room temperature; PS—rats were orally gavaged with PBS and exposed to heat stress; PC—rats were orally gavaged with PBS and kept at room temperature; *P < 0·05.

Analysis of the gut microbial community in rats from different experimental groups revealed significant decrease of anaerobic to aerobic bacteria ratio in rats from PS group in comparison with all other groups. No difference in this ratio was found in rats, treated with EH (Fig. 4a). Significant elevation of *Escherichia* spp. (Fig. 4b), haemolytic bacteria (Fig. 4c) and *Staphylococcus* spp. (Fig. 4d) was found in rats from PS group. Number of *Staphylococcus* spp. and haemolytic bacteria was significantly higher in animals from ES group in comparison with PC group, but significantly lower than in animals from PS group. No difference in *Bifidobacterium* spp. number was observed in groups of animals, pretreated with PBS (PC, PS), but treatment with EH resulted in significant elevation of these bacteria (groups ES, EC) (Fig. 4e). The highest number of *Lactobacullus* spp. was revealed in rats pretreated with PBS before exposure to heat stress conditions (Fig4f). Treatment with EH did not affect *Lactobacillus* spp. number.

### 16S rRNA sequencing of the gut microbiota

**Figure 5:**
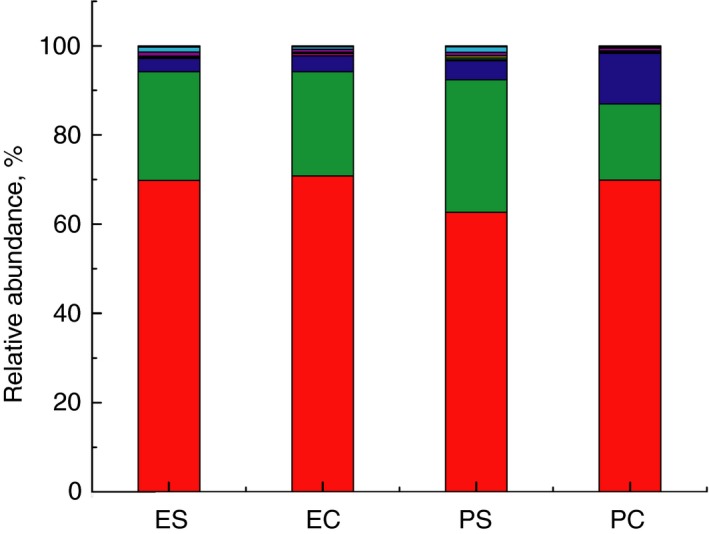
Composition of the gut microbiota of rats from different experimental groups at the phylum level. All phyla present in abundance of <0·1% are included as other. ES—rats were orally gavaged with *Saccharomyces cerevisiae* fermentate and exposed to heat stress; EC—rats were orally gavaged with *Saccharomyces cerevisiae* fermentate and kept at room temperature; PS—rats were orally gavaged with PBS and exposed to heat stress; PC—rats were orally gavaged with PBS and kept at room temperature ((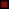) other; (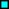) *Verrucomicrobia*; (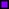) *Tenericutes*; (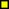) TM‐7; (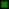) *Deferribacteres*; (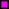) *Proteobacteria*; (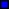) *Actinobacteria*; (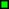) *Bacteriodetes*; (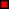) Firmicutes). [Colour figure can be viewed at http://wileyonlinelibrary.com]

After stringent quality sequence curation, a total of 1 565 513 sequences were parsed and 1 382 946 were then clustered. 1 382 796 sequences identified within the *Bacteria* and *Archaea* domains were utilized for final microbiota analyses. The average reads per sample was 60 121. Ten different phyla were identified. The most abundant phyla in the gut microbiota of rats from different experimental groups were *Firmicutes, Bacteroidetes* and *Actinobacteria* (Fig. 5). *Firmicutes* was a dominant phylum (68·3%) followed by *Bacteroidetes* (23·6%) and *Actinobacteria* (5·5%). Significantly higher number of *Actinobacteria* was found in PC group (11·3 ± 1·8%, *P* < 0·05), *Bacteroidetes* were prevalent in PS group (29·7 ± 4·8%, *P* < 0·05).

**Figure 6:**
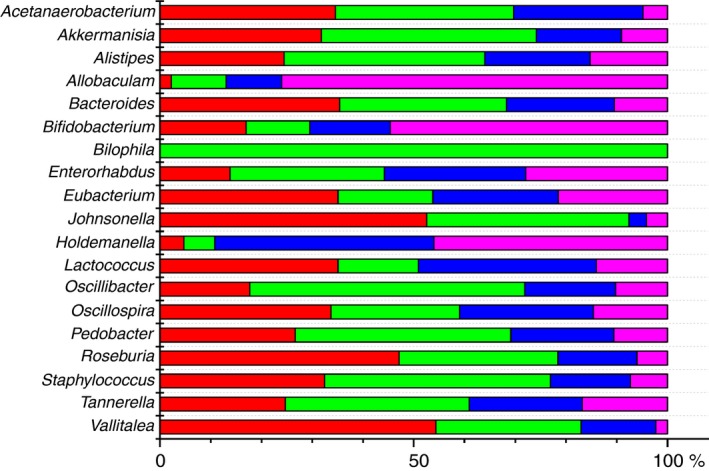
Microbial composition of the gut microbiota in different groups is presented as a per cent of abundance. (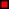) ES—rats were orally gavaged with *Saccharomyces cerevisiae* fermentate and exposed to heat stress; (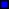) EC—rats were orally gavaged with *Saccharomyces cerevisiae* fermentate and kept at room temperature; (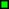) PS—rats were orally gavaged with PBS and exposed to heat stress; (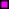) PC—rats were orally gavaged with PBS and kept at room temperature. [Colour figure can be viewed at http://wileyonlinelibrary.com]

At the genus level the most significant changes were found in PS group in comparison with control PC group (Fig. 6; Table 1). Totally 14 genera were affected by heat stress. Some genera considerably increased (*Acetanaerobacterium, Akkermansia, Allistipes, Allobaculum, Bacteroides, Johnsonella, Oscillibacter, Staphylococcus, Tannerella),* whereas others (*Bifidobacterium, Enterorhabdus, Holdemanella, Pedobacter*) significantly decreased. *Bilophila* was absent in rats from PC group, but detected in PS rats. Treatment of rats with yeast fermentate before exposure to heat stress (ES group) resulted in less changes of gut microbiota. Only nine genera were significantly changed: relative abundance of *Bifidobacterium* and *Allobaculum* were declined, while *Acetanaerobacterium, Bacteroides, Eubacterium, Johnsonella, Lactococcus, Oscillospira, Roseburia and Vallitalea,* substantially increased. *Akkermansia* and *Staphylococcus* were significantly higher only in rats from PS group in comparison with animals from PC group. Minor changes in the gut microbiota were found in EC group of rats in comparison with PC group—only *Bifidobacterium* significantly decreased.

**Table 1:** Changes in the gut microbiota genera after different treatments

Genera	PC		PS		PS *vs* PC changes, %	*P* value
	Mean	SEM	Mean	SEM		
*Acetanaerobacterium*	0·0035	0·0009	0·0252	0·0061	619·4603	0·0053
*Akkermansia*	0·3169	0·1184	1·4911	0·4477	370·1025	0·0219
*Alistipes*	2·8117	0·5532	7·2977	1·5431	159·7039	0·0183
*Allobaculum*	4·7639	1·2169	0·5713	0·4029	−88·0252	0·0113
*Bacteroides*	0·8846	0·1283	2·7718	0·5332	214·7727	0·0063
*Bifidobacterium*	10·6204	0·6910	2·4397	0·8783	−77·0245	0·0015
*Bilophila*	0	0	0·0016	0·0007	PS*	
*Enterorhabdus*	0·0537	0·0102	0·0265	0·0034	−50·5617	0·0302
*Johnsonella*	0·0007	0·0003	0·0081	0·0036	995·8904	0·0404
*Holdemanella*	3·5545	0·8240	0·4755	0·2157	−86·6236	0·0047
*Oscillibacter*	0·0071	0·0026	0·0352	0·0108	402·4449	0·0274
*Pedobacter*	0·0623	0·0021	0·0241	0·0052	−59·7735	0·0094
*Tannerella*	0·0932	0·0178	0·2137	0·0243	137·5029	0·0034
*Staphylococcus*	0·3586	0·0809	2·2036	0·7185	514·4869	0·0341
						

Genera	PC		ES		ES *vs* PC Changes, %	*P* value
	Mean	SEM	Mean	SEM		
*Acetanaerobacterium*	0·0035	0·0009	0·0248	0·0081	615·9931	0·0258
*Allobaculum*	4·7639	1·2169	0·2186	0·0691	−95·4110	0·0257
*Bacteroides*	0·8846	0·1283	2·9801	0·4812	236·8742	0·0018
*Bifidobacterium*	10·6204	0·6910	3·2922	1·7735	−69·0207	0·0026
*Eubacterium*	4·0526	0·5370	6·5942	0·6038	62·7175	0·0104
*Johnsonella*	0·0007	0·0003	0·0092	0·0014	1156·9830	0·0001
*Lactococcus*	0·0050	0·0016	0·1045	0·0415	214·1153	0·0437
*Oscillospira*	0·9808	0·1301	2·2579	0·3157	130·2043	0·0038
*Roseburia*	0·0268	0·0049	0·2091	0·0471	679·2159	0·0032
*Vallitalea*	0·0005	0·0003	0·0114	0·0038	2231·2421	0·0169
						

Genera	PC		EC		EC *vs* PC Changes, %	*P* value
	Mean	SEM	Mean	SEM		
*Bifidobacterium*	10·6204	0·6910	3·0878	0·9778	−70·9260	0·0001

PC—rats were pretreated with PBS and kept at room temperature; PS—rats were pretreated with PBS and exposed to heat stress; ES—rats were pretreated with *Saccharomyces cerevisiae* fermentate and exposed to heat stress; EC—rats were pretreated with *Saccharomyces cerevisiae* fermentate and kept at room temperature.

* Genus was found only in this group.

## Discussion

This study aimed to evaluate the efficacy of the *S. cerevisiae* fermentate in protection of the intestinal barrier function and in modulating the gut microbiota during heat stress. Exposure of rats, pretreated with PBS, to heat stress conditions resulted in significant decrease of occludin, claudin, ZO‐1 and JAM‐A expression. Decreased expression of TJ proteins during heat stress was found in Caco‐2 cells (Gupta *et al.,* 2017) and in animal studies (Wu *et al.,* 2018). Inhibition of these proteins expression indicates the disturbance of the TJ barrier functions and accompanied by intestinal permeability (He *et al.,* 2016). Our results showed that oral administration of *S. cerevisiae* fermentate to rats before heat stress significantly enhanced TJ proteins expression. In previous studies, this fermentate demonstrated prebiotic activity by protection against inflammation (Possemiers *et al.,* 2013) and improvement of gastrointestinal discomfort in patients (Pinheiro *et al.,* 2017). The findings of other authors revealed a positive role of prebiotics in supporting of normal intestinal barrier function. Thus, the dietary use of inulin‐enriched pasta by healthy volunteers protected intestinal barrier functioning during physical exercise (Russo *et al.,* 2012). Cani *et al. *(2009) found that oligofructose‐enriched diet contributed to the improvement of gut barrier function in obese mice by up‐regulation of TJ proteins expression.

We observed that heat stress resulted in significant decrease of Paneth and goblet cells in the intestine of rats. Paneth and goblet cells are essential components of the intestinal epithelium and contribute to the barrier function of epithelium (Furness *et al.,* 2013). Depletion of these cells may lead to the development of an epithelial barrier defect (Estienne *et al.,* 2010). Reduction of Paneth and goblet cells was shown to increase sensitivity of mice to TNF‐induced toxicity, accompanied by increased hypothermia, lethality and intestinal permeability (Van Hauwermeiren *et al.,* 2015). Decrease of these cells was induced by different stress conditions, such as neonatal maternal separation (Bessette *et al.,* 2016), chronic and heat stress (Deng *et al.,* 2012; Gao *et al.,* 2018). Our results revealed that pretreatment of rats with *S. cerevisiae* fermentate before exposure to heat stress prevented decline of Paneth and goblet cells. Beneficial effect of cell wall from *S. cerevisiae* as a dietary supplement for stabilization of goblet cells in chickens was demonstrated by Baurhoo *et al. *(2009). Prebiotic inulin in combination with rutin reduced inflammatory status and endoplasmic reticulum stress in Paneth cells (Guo *et al.,* 2018).

Paneth and goblet cells are indispensable for maintaining homeostasis with enteric microbes (Baurhoo *et al.,* 2007; Vaishnava *et al.,* 2008) as they promote the removal of microbes from the mucosal surface (Chairatana and Nolan, 2017). Reduction in number or defects in activity of these cells lead to microbiota disbiosis (Baurhoo *et al.,* 2007; Riba *et al.,* 2017). Our data showed significant changes in the gut microbiota only in rats from PS group with substantial depletion of Paneth and goblet cells. Thus, culture‐based bacteriological analysis of the gut microbiota revealed decrease of anaerobic to aerobic bacteria ratio in these animals. It is well known that most microorganisms in the distal small intestine and colon are anaerobes (Weng and Walker, 2013), which numerously exceed aerobic bacteria in the gut (Maity *et al.,* 2012). The predominance of aerobic bacteria in the gut microbiota has been found in the patients with colon cancer (Vargo *et al.,* 1980), necrotizing fasciitis (Saini *et al.,* 2004), in malnutrition (Million *et al.,* 2016) and in severely burned patients (Chen *et al.,* 1998) indicating an imbalance of the intestinal microbiota. We also found significant increase of haemolytic bacteria, *Escherichia* spp. and *Staphylococcus* spp. in rats of PS group. Elevated number of bacteria with haemolytic activity indicates the microbiota disorder (Popova *et al.,* 2017) as these bacteria can be a potentiator of intestinal inflammation and epithelial dysfunction in the gut (Wiegand *et al.,* 2017). Imbalance in quantitative composition of *Escherichia* spp. and *Staphylococcus* spp. also specifies dysbiotic changes of the gut microbiota (Popova *et al.,* 2017; Itani *et al.,* 2018). The number of *Lactobacillus* spp. was significantly higher in rats of PS group in comparison with other groups of animals. The effect of stress on lactobacilli in the gut is estimated differently by researchers. Some of them observed an increase of *Lactobacillus* spp. during chronic stress (Wong *et al.,* 2016), while others reported about depleting of these bacteria in stressed animals (Marin *et al.,* 2017). Treatment with EH did not change the relative abundance of *Lactobacillus* spp. The same result was obtained with EH in clinical trial (Pinheiro *et al.,* 2017). We did not find the difference in *Bifidobacterium* spp. number in groups of rats pretreated with PBS (PS and PC groups). But administration of EH significantly increased the number of bifidobacteria. Positive effect of EH on *Bifidobacterium* was previously observed *in vitro* study (Possemiers *et al.,* 2013). Stimulation of bifidobacteria in the gut of elderly people by prebiotic supplementation was found in clinical trials (Guigoz *et al.,* 2002).

High‐throughput 16S rRNA gene sequencing revealed that in all groups of rats *Firmicutes* was a dominant phylum that is in accordance with the data of other authors (Golubeva *et al.,* 2015; Byerley *et al.,* 2017). Significant changes of the gut microbiota in different groups were found at the genus taxonomic level. Exposure of rats to heat stress conditions (PS group) resulted in substantial decrease of beneficial bacteria (*Allobaculum, Bifidobacterium*) in comparison with control (PC) group. Beneficial effects of these bacteria were shown in many studies. Thus, *Allobaculum* was associated with prevention of obesity and insulin resistance (Everard *et al.,* 2014), *Bifidobacterium* are known as a normal component of the gut microbiota and as probiotics for human and animal consumption (Russell *et al.,* 2011). *Enterorhabdus* and *Pedobacter* were also decreased in PS group of rats. *Enterorhabdus* was shown to be associated with autism spectrum disorder in a murine model (de Theije *et al.,* 2014) and with a genetic variant of the human leukocyte antigen complex that has been related to inflammatory diseases (Opstelten *et al.,* 2016). *Pedobacter,* heparinase‐produsing bacteria, are a normal component of the gut microbiota of healthy fish (Wang *et al.,* 2018a) and the medicinal leech (Ott *et al.,* 2015). Significant increase of pathogenic bacteria (*Alistipes, Bacteroides, Bilophila, Johnsonella, Oscillibacter, Tannerella* and *Staphylococcus)* was found in PS group. This result corresponds to our data from the culture‐based analysis of the microbiota, testifying that elevation of pathogenic bacteria was observed only in rats from PS group. *Alistipes, Bacteroides* and *Bilophila* were overrepresented in the carcinoma patients (Feng *et al.,* 2015). *Bilophila* is one of the most common anaerobic bacteria recovered from patients with perforated and gangrenous appendicitis (Baron, 1997). It was shown, that increased number of *Bilophila* induces systemic inflammation and contribute to the commencement of the chronic diseases (Feng *et al.,* 2017). *Johnsonella* was highly associated with tumour site (Pushalkar *et al.,* 2012) and with chronic obstructive pulmonary disease (Wu *et al.,* 2017), *Tannerella* was found to be a predisposing factor in atherosclerosis progression (Lee *et al.,* 2014). Our data show that stress results in significant increase of *Oscillibacter*, which is known as a potential opportunistic pathogen, positively correlated with gut permeability (Lam *et al.,* 2012). We hypothesize that *Oscillibacter* bacteria could be related to the disturbance in the TJ proteins expression, observed in PS group. Two genera (*Acetanaerobacterium* and *Akkermansia*) were elevated after heat stress. There are some evidence of beneficial effects of *Acetanaerobacterium,* associated with the high production of enterolactone (Hullar *et al.,* 2015), which may protect against hormone‐dependent cancers and cardiovascular diseases (Kilkkinen *et al.,* 2001). *Akkermansia muciniphila* is a mucin‐degrading bacterium, considered by some authors as an important member of the gut microbiota for control of physiological and homeostatic functions during obesity and type 2 diabetes (Everard *et al.,* 2013). Conversely, other studies showed that increased abundance of *A. muciniphila* is related to hypertension (Tain *et al.,* 2018) and can impair intestinal barrier function after using mucin by these bacteria as a nutrient (Desai *et al.,* 2016). Depletion of the mucus layer by enriched *A. muciniphila* was associated with higher susceptibility to a gastrointestinal pathogen. Analysis of the microbiota in PS group indicates that disturbance in the microbial community is mostly by increase of pathogenic bacteria. Our results revealed that *Akkermansia* number was considerably higher only in PS group, where intestinal barrier function was disrupted. Previously we showed that exposure of rats to heat stress conditions significantly decreases the total thickness of intestinal mucosa (Ducray *et al.,* 2016). Treatment with EH before stress (group ES) prevented increase of *Akkermansia* and destruction of intestinal barrier. These results are consistent with data from Desai *et al. *(2016), who found that abundance of *A. muciniphila* increased rapidly in the absence of prebiotic. We found significant decrease of two genera (*Allobaculum, Bifidobacterium*) and increase of *Acetanaerobacterium, Bacteroides, Johnsonella* in microbiota of rats from ES group *vs* PC group. Same trend presents in PS group that indicates specific effect of stress on these groups of bacteria. Essential impact of the EH on microbiota during heat stress is manifested in elevated number of beneficial bacteria (*Eubacterium, Lactococcus, Oscillospira, Roseburia, Vallitalea). Roseburia*, *Eubacterium* and *Oscillospira* are butyrate‐producing bacterial genera, positively correlate with antioxidant activities and negatively correlate with inflammation (Gophna *et al.,* 2017; Wang *et al.,* 2018b). Our results are consistent with previously *in vitro* study of Possemiers *et al. *(2013), who showed that yeast fermentate induces butyrate production and possess anti‐inflammatory activity. Butyrate is recognized as an essential host energy source (Donohoe *et al.,* 2011), which can protect the mucus layer from injury (van der Beek *et al.,* 2017). Positive contribution of *Lactococcus* and *Vallitalea* to the change of microbiota was noticed by other authors in humans and animals (Borrelli *et al.,* 2017; Savage *et al.,* 2018; Mao *et al.,* 2018). We did not find significant change of the microbiota in EC group, except decreased abundance of *Bifidobacterium*. Data about lower number of *Bifidobacterium* in PS, ES and EC groups are in contrast with culture‐based results. Other authors also reported that species, isolated from culture did not generally correspond with the most abundant genera in microbiome analysis (Koeller *et al.,* 2018). For example, increased *Bifidobacterium* abundance was detectable only with an *in vitro* culture method, and not pyrosequencing (Finegold *et al.,* 2014). It was shown that the abundance of *Bifidobacterium* in humans and animals is underestimated with 16S rRNA gene‐based approach (Hooda *et al.,* 2012).

Our results revealed substantial effect of *S. cerevisiae* fermentate prebiotic in prevention of heat stress‐related complications. Oral treatment of rats with prebiotic before exposure to heat stress conditions protected disruption of Paneth and goblet cells homeostasis, maintained expression of TJ proteins. We suggest that these effects are associated with beneficial modulation of the gut microbiota by prebiotic.

## Conflict of Interest

No conflict of interest declared.
